# Seasonal shedding of coronavirus by straw-colored fruit bats at urban roosts in Africa

**DOI:** 10.1371/journal.pone.0274490

**Published:** 2022-09-15

**Authors:** Diego Montecino-Latorre, Tracey Goldstein, Terra R. Kelly, David J. Wolking, Adam Kindunda, Godphrey Kongo, Samuel O. Bel-Nono, Rudovick R. Kazwala, Richard D. Suu-Ire, Christopher M. Barker, Christine Kreuder Johnson, Jonna A. K. Mazet

**Affiliations:** 1 One Health Institute, School of Veterinary Medicine, University of California, Davis, California, United States of America; 2 Department of Pathology, Microbiology, and Immunology, School of Veterinary Medicine, University of California, Davis, California, United States of America; 3 College of Veterinary Medicine and Biomedical Sciences, Sokoine University of Agriculture, Morogoro, Tanzania; 4 PREDICT Project, Ghana; 5 School of Veterinary Medicine, College of Basic and Applied Sciences, University of Ghana, Legon, Accra, Ghana; University of Reunion Island, RÉUNION

## Abstract

The straw-colored fruit bat (*Eidolon helvum*) is a pteropodid whose conservation is crucial for maintaining functional connectivity of plant populations in tropical Africa. Land conversion has pushed this species to adapt to roosting in urban centers across its range. These colonies often host millions of individuals, creating intensive human-bat contact interfaces that could facilitate the spillover of coronaviruses shed by these bats. A better understanding of coronavirus dynamics in these roosts is needed to identify peak times of exposure risk in order to propose evidence-based management that supports safe human-bat coexistence, as well as the conservation of this chiropteran. We studied the temporal patterns of coronavirus shedding in *E*. *helvum*, by testing thousands of longitudinally-collected fecal samples from two spatially distant urban roosts in Ghana and Tanzania. Shedding of coronaviruses peaked during the second part of pup weaning in both roosts. Assuming that coronavirus shedding is directly related to spillover risk, our results indicate that exposure mitigation should target reducing contact between people and *E*. *helvum* roosts during the pup “weaning” period. This recommendation can be applied across the many highly-populated urban sites occupied by *E*. *helvum* across Africa.

## Introduction

The straw-colored fruit bat (Eidolon helvum) is a pteropodid widely distributed in tropical Africa with a described range of at least ~12 million km2 [1, 2]. Its reproductive cycle is seasonal, with delayed implantation [3] and a highly synchronized annual birth pulse [4–6]. Estrus, pregnancy, and birthing seem to occur in several locations but their timing can vary across latitudes [4–8]. *E*. *helvum* bats roost in trees, forming dynamic colonies that can host millions of individuals [5, 9–11], and massive migration can cause a 40–50 fold roost size difference between the annual minimum and peak roost size [11–14].

*E*. *helvum* is thought to be a unique seed disperser as a consequence of its large migratory movements and variable gut passage time [15–17]. Given the ongoing habitat fragmentation in Africa, seed dispersers that travel over large distances and retain seeds for long periods are particularly important for maintaining functional connectivity and gene flow of plant populations in degraded landscapes [16]. Consequently, the conservation of this bat species and the provision of its ecosystem services are crucial for tropical Africa.

The straw-colored fruit bat is also a distinct species because it has adapted to habitat destruction by occupying trees in several busy urban centers across its range [7, 18, 19]. These roosts can host millions of individuals [11, 12], creating intensive human-bat contact interfaces that facilitate exposure of people to the feces and urine of these chiropterans [7, 20] and possibly to pathogens that are shed through these excretions.

Indeed, available data suggest that *E*. *helvum* hosts several viruses. Surveys in urban and non-urban colonies have reported the detection of viral RNA and DNA and isolation of viruses from diverse viral taxonomic families including those with zoonotic species ([19, 21–23]; S1 File). Coronaviruses (Coronaviridae family; CoVs) are an important group of viruses previously detected in these bats [24–32]. This viral family includes SARS-CoV-1, MERS CoV, the etiology of the current COVID-19 pandemic (SARS-CoV-2) and around half of the new viruses with highest ranking of animal to human transmission risk following expert opinion [33].

Concerns about public health risks associated with prevalent CoVs and intense human-bat interfaces across the extended range of *E*. *helvum*, together with the strong need to support the conservation of this chiropteran, underscore the management challenge. Better understanding of CoV dynamics in urban roosts are needed to propose evidence-based risk management that promotes the safe coexistence of *E*. *helvum* and people. If viral shedding has high and low periods, then viral exposure could be mitigated through seasonal management of human behavior. However, CoV shedding patterns in *E*. *helvum* roosts remain almost unknown. Indeed, there is a lack of general understanding of CoV ecology in African bats, despite these viruses having been reported in these chiropterans [34, 35] and areas of Africa having been identified as global hot spots of disease emergence [36].

Here, we aimed to identify CoV shedding patterns in *E*. *helvum* roosts and propose data-based realistic strategies that could support a safer, ethical coexistence between bats and people. To accomplish these objectives, we conducted a unique, robust, longitudinal collection and testing of thousands of fecal samples for an entire year in two spatially distant roosts (Ghana and Tanzania, located ~4,400 km apart). We hypothesized that CoV shedding in these roosts is variable over time and associated with *E*. *helvum* annual reproductive events. The identification of such events could offer opportunities for targeted management to reduce human exposure risk at the studied roosts and others across Africa. We tested this hypothesis by comparing the fit of different logistic models to the data and by estimating the association between the reproductive periods and CoV shedding.

## Methods

### Study period and studied roosts

**Fig 1:**
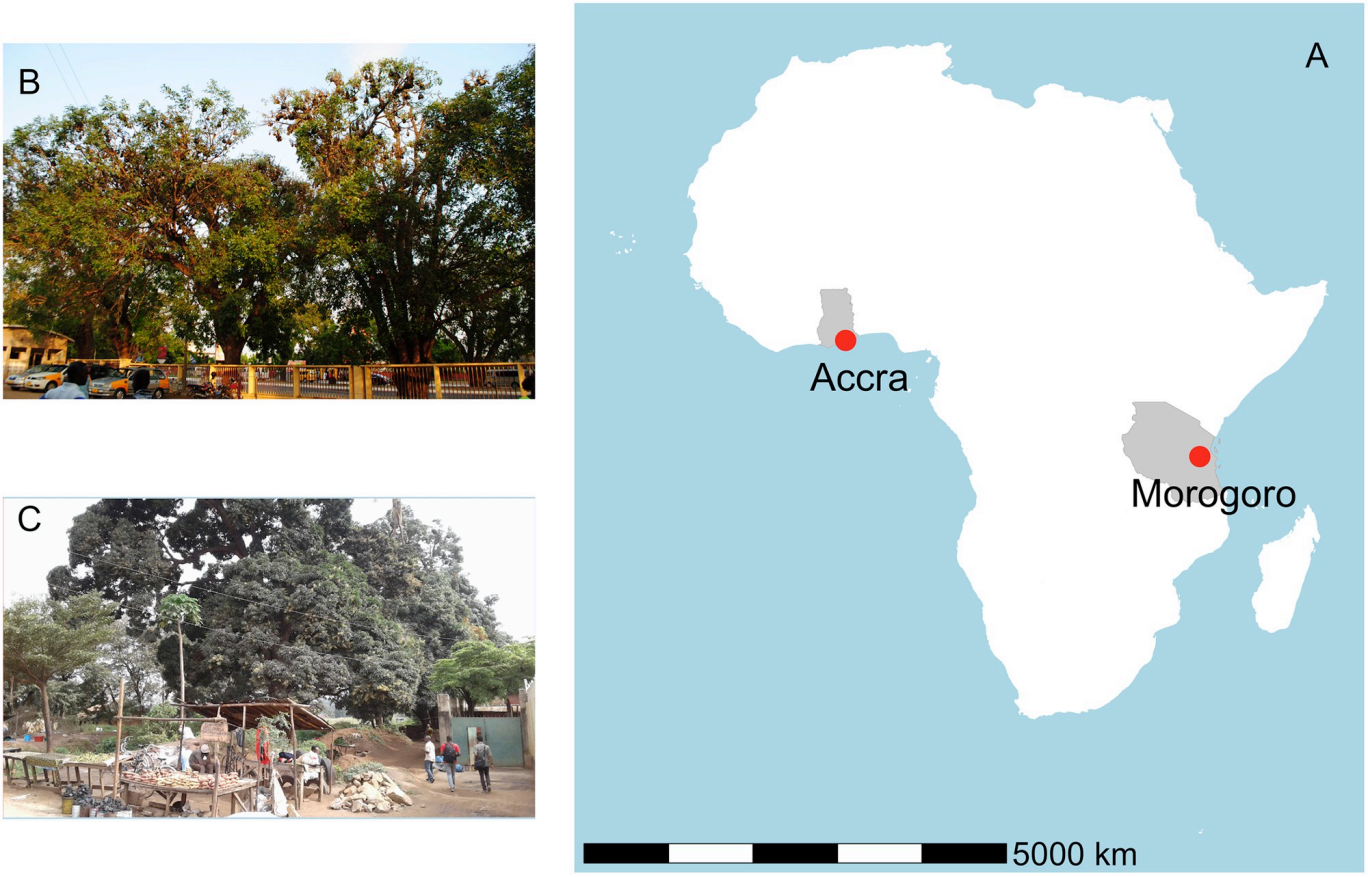
Location of the studied *Eidolon helvum* roosts. Panel A shows the locations of the roosts in Africa. Panel B shows some of the trees occupied at the 37 Military Hospital in Accra, Ghana and Panel C shows roosting bats at the Kikundi Market in Morogoro, Tanzania.

We studied two previously described *E*. *helvum* urban colonies: the roost at the 37 Military Hospital (5.5882, -0.1824) in Accra, Ghana (West Africa; [7, 11, 19, 21, 37, 38]) and at the Kikundi Market and Nunge Court (-6.8233, 37.6662) in Morogoro, Tanzania (East Africa; [7]; Fig 1). Accra is the capital city of the Republic of Ghana with a population of 2.9 million people and Morogoro is a regional capital in Eastern Tanzania with a population of 315,866 people [39, 40]. The 37 Military Hospital is located in a busy and heavily urbanized area of Accra, at the junction of two main avenues with major car, public transport, and pedestrian traffic. Occupied trees are within and around the hospital property. The Kikundi Market and Nunge Court are situated in a busy commercial area of Morogoro mainly occupied by pedestrians and market vendors. Human structures at this site are not as developed nor dense compared to the 37 Military Hospital area. Occupied trees are adjacent to public buildings and houses, as well as empty plots. The 37 Military Hospital roost in Accra (hereafter “Accra”) was studied during March 2017-February 2018. The Kikundi Market and Nunge Court roost in Morogoro (hereafter “Morogoro”) was studied during August 2017-July 2018. These roosts are separated by ~4,400 km (Fig 1).

### Roost census

We counted the straw-colored fruit bats in both colonies on a monthly basis over an entire year during the study period (see above). We followed a previously applied method to obtain an index of abundance of the *E*. *helvum* population (e.g., [12]). Briefly, we censused bats in the colonies by hierarchically adding the estimated number of bats per roosting group (cluster), per tree branch, and per tree.

The censuses were conducted during the morning when the bats settled down after foraging. Broad-canopy trees allowed the counting of bats in all occupied trees. No other bat species were observed, and barcoding of a subset of fecal samples corroborated the single species composition.

### Roost sampling

We collected 97 fecal samples per roost per month, based on an expected CoV shedding proportion of 0.1, an expected precision of 0.1, and a confidence level of 95% (Z = 1.96). Each month, we set eight new 16 m^2^ plastic sheets (four by four m disposable tablecloths) below a specific set of trees to collect feces as previously described ([21, 41]; S1 Fig). In Accra, the locations of the plastic sheets were consistent across monthly sampling events. In Morogoro, the locations of the plastic sheet placement varied as the colony moved between the Kikundi Market and the nearby Nunge Court during the study period (see Results section). However, within each area, the locations of the plastic sheets were consistent.

Feces were collected between 3:30 and 6:30 am in order to catch samples from the bats as they were returning to the roost, obtain fresh droppings deposited beneath the trees, and optimize timing when fewer people were present at the sites. We assumed that each fecal sample belonged to a single bat, as they typically defecate once during this sampling time period [16] and because we collected samples ~1 m apart from each other. Samples were first collected along the edges of the first plastic sheet (~12 samples) followed by sample collection from the central area (~4 more samples). Then we moved to the next plastic sheet and repeated the process.

Feces were collected using a sterile polyester-tipped swab as they were deposited to limit contamination with urine and feces from other bats. Specimens (swab tip and feces) were placed into separate tubes containing either Trizol^®^ reagent or Viral Transport Media, immediately stored in liquid nitrogen, and kept at -80 C until testing.

Non-invasive sample collection and exportation of samples to the United States was accomplished with the permission of the Tanzania Research Institute, the Wildlife Division of the Ghana Forestry Commission (2017-264-ER-2011-29), the Ghana Veterinary Services Directorate of the Ministry of Food and Agriculture, the Noguchi Memorial Institute for Medical Research, University of Ghana (2016-01-1X), and the Institutional Animal Care and Use Committees (IACUC) at the University of California, Davis (protocol number: 16048).

### Coronavirus presence and viral identification

We extracted RNA from all fecal samples (2,328 total samples), and cDNA was prepared as previously described [34]. Two broadly reactive consensus PCR assays (“Quan” and “Watanabe”) targeting different peptides of the RNA-dependent RNA polymerase were used in order to detect both known and novel coronaviruses [42, 43]. Amplified products of the expected size (a ~332 bp length fragment for Quan and a ~434 bp length fragment for Watanabe) were cloned and sequenced as reported by Anthony et al. [34]. We conducted a BLAST analysis to compare the nucleotide sequences to existing CoV RNA-dependent RNA-polymerase gene sequences in the GenBank database using Geneious Prime 2019.2 [44]**.** Sequences were taxonomically classified following Anthony et al. [34]. A fecal sample was considered positive when at least one assay was PCR-positive and it was taxonomically classified as a CoV. We assumed that a bat sourcing a positive fecal sample was shedding CoVs.

### Reproductive cycle

We did not capture bats for this study; therefore, direct confirmation of pregnant females, neonates, and juveniles was not feasible. Moreover, we did not identify nor count pups attached to dams because they are very difficult to observe [1, 7, 11]. Consequently, the reproductive cycle was inferred based on: i) our previous data of this species in Morogoro and Accra; ii) the synchronized yearly birth pulse of this species [3, 5, 18, 45]; iii) the potential heterogeneity in the time of estrus, pregnancy, and birth pulse across latitudes [4, 5, 45]; iv) the previous observation of neonates in Ghana [38]; v) previous observations in the Morogoro and Accra roosts [7, 8, 11, 19, 37, 46]; and vi) reported birth pulse of 2.5–3 months and 60 days of lactation period [18].

Therefore, in the case of Accra, we assigned April 15th as the start date of the “lactation” period, June 15th as the last day of the birth pulse, and June 16th as the first date of the pup weaning period. In the case of Morogoro, we assigned December 15th as the start date of the “lactation” period, February 15th as the last day of the birth pulse, and February 16th as the first date of the “weaning” period. The end of the weaning period was set four months later (October 15th and June 15th in Accra and Morogoro, respectively) because we expected the potential effects of the “weaning” on CoV dynamics to last up to one month after the last pups of the season were weaned. The days immediately following these dates were set as the beginning of the “rest of year” period in the corresponding colonies. The defined reproductive seasons were consistent with previously proposed reproductive cycles [4, 7, 18, 45].

### Precipitation

Daily cumulative precipitation per month was quantified using data from the ERA-interim, a global atmospheric reanalysis at 0.75° x 0.75° resolution (T255) gridded climatic database from the European Centre for Medium-Range Weather Forecasts [47]. We tracked the precipitation during the study period and assessed the appropriateness of the inferred reproductive cycle. Specifically, we expected the precipitation peak to occur after the birth pulse as reported in other *E*. *helvum* East- and West-African roosts [18, 45].

### Statistical analysis

To assess the seasonality of CoV shedding, we compared an “intercept-only” logistic model that assumed constant CoV shedding over time versus a “sine-cosine” logistic model that allowed for seasonal cycling of CoV shedding with a period of 12 months and a single annual maximum and minimum [48]. Shedding of CoV in the *i*^th^ *E*. *helvum* fecal samples was modeled as a *Bernoulli* process (S2 File). The fit of these logistic models to the data was compared using the Leave-one-out information criterion (LOOIC [49]) as implemented in the package “loo” for R [50]. We considered that seasonality was supported if the sine-cosine model had a better fit (lower LOOIC) and if the 95% Highest Posterior Density Intervals (95% HPDIs) of at least two monthly CoV shedding posterior predictive distributions (PpreDs) did not overlap. The 95% HPDI shows the narrowest interval of values containing the 95% of a distribution’s density. Because few positives were detected in Accra, statistical modeling was conducted with Morogoro data only.

To assess the association between the reproductive cycle and CoV shedding, we constructed two logistic models. The “fixed effects” model linked the detection of CoV, assumed as a *Bernoulli* process, to the reproductive periods. The second model, the “hierarchical model”, also assumed a *Bernoulli* process but grouped *E*. *helvum* fecal samples per month of collection, and months were grouped per reproductive period (S2 File). The “fixed effects” model supported that bats have higher odds of CoV shedding during the “weaning” period compared to the “rest of the year” if the 95% HPDI of the corresponding odds ratio Posterior Probability Distributions (PProD) did not include 1. Similarly, we evaluated the odds of CoV shedding during the “lactation” versus the “rest of the year” periods and during the “weaning” versus the “lactation” periods. The “hierarchical model” supported that the odds of a CoV shedding during month *m* belonging to the *r* reproductive period were higher than the odds in month *m*’ within the reproductive period *r*’ if the 95% HPDI of the corresponding odds ratio PProD did not include 1. Moreover, a larger standard deviation of the reproductive period distribution (σ_R_) compared to a larger standard deviation of the month distribution (σ_M_) suggested larger CoV shedding variability among reproductive periods than among months.

All models were constructed using Stan v. 2.17.0 which was run from R v. 3.6.0 through the package RStan v. 2.18.2 [51–53]. More details of the Markov Chain Monte Carlo (MCMC) sampling and sampling diagnostics are provided in S3 File.

## Results

### Roost census

**Fig 2:**
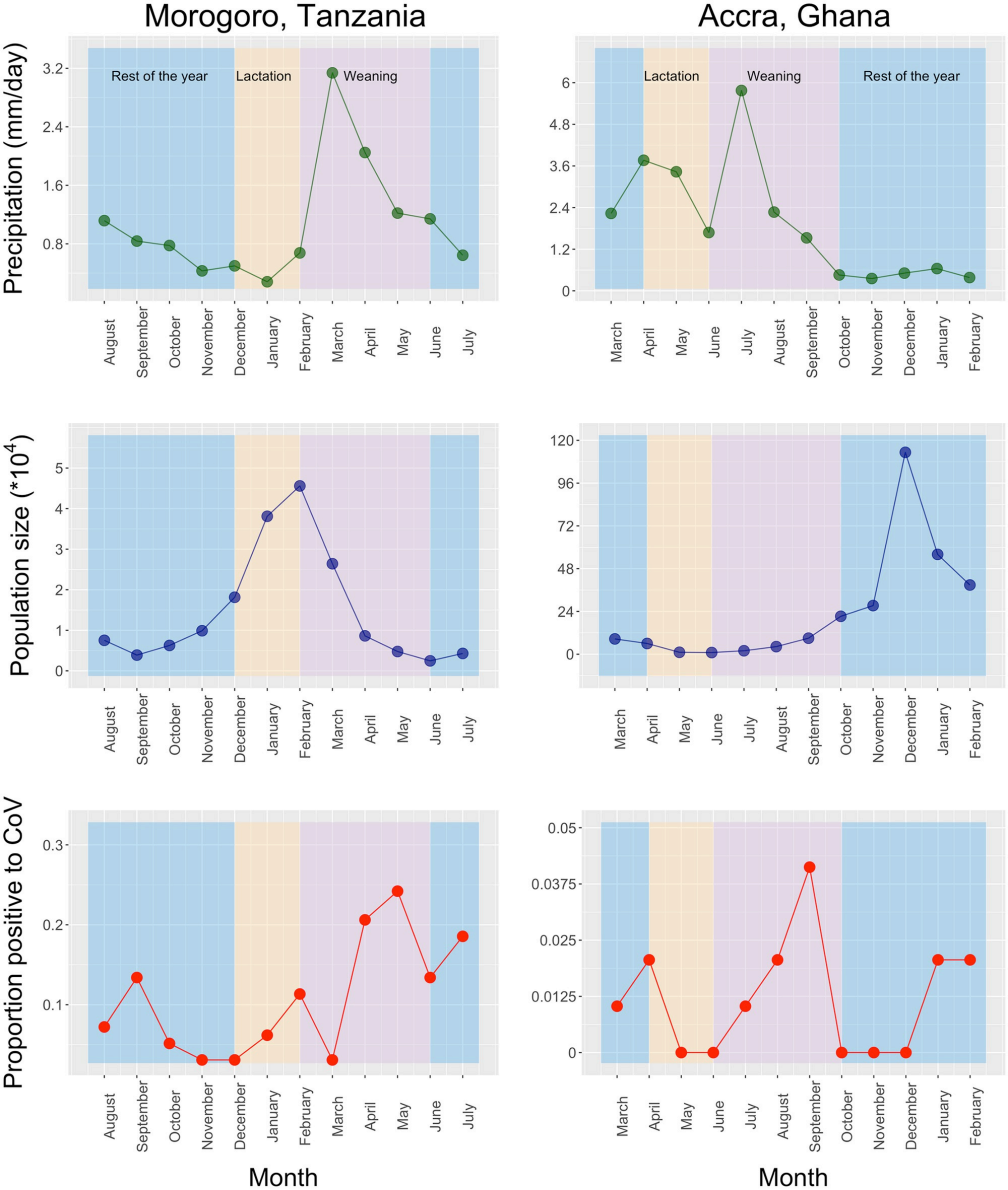
*Eidolon helvum* monthly abundance (blue line), precipitation (green line), and coronavirus shedding (red line) at the roost in Morogoro, Tanzania (left); and at the roost in Accra, Ghana (right). Color bands indicate the “lactation” (orange), “weaning” (purple), and “rest of the year” (blue) reproductive periods.

During the 12-month study period, the Morogoro colony roosted at the Kikundi Market from August to October 2017 and from May to June 2018. The colony was settled in Nunge Court from November 2017 to April 2018, and in July 2018. These locations are separated by 850 m. Morogoro roost abundance peaked during December 2017-March 2018 (~45,000 in February) and was smallest from August to November 2017 and April to July 2018 (~2,500 in June). The Accra roost peaked during December 2017-February 2018 (~1.2 million in December) and reached a nadir from March to November 2017 (~4,000 in June). Bat abundance in Morogoro increased during the “lactation” period, peaked at the beginning of the “weaning” period, and began to decline after the wet season began. Bat abundance in Accra remained relatively constant during the “lactation” and “weaning” periods, increased steeply until midpoint of the “rest of the year” period, and decreased thereafter (Fig 2). We did not count pups attached to the dams; therefore, the counts reflected migration and seasonal addition of weaned pups.

### Roost sampling and coronavirus identification

Overall, 125 and 14 fecal samples were positive for CoVs in Morogoro and Accra, respectively (proportion of positive samples were 0.107 [125/1164] and 0.012 [14/1164], respectively). The Genbank Accession Numbers are MT797294.1-MT797304.1; MT797562.1-MT797572.1; MT797305.1-MT797384.1; MT797573.1-MT797628.1; and MW007350.1. The monthly proportion of positive feces varied with a minimum of 0.03 and 0 and peaks of 0.24 and 0.04 in Morogoro and Accra, respectively. The proportion of positive feces in the “lactation”, “weaning”, and the “rest of the year” periods were 0.088, 0.153, and 0.084 in Morogoro and 0, 0.018, and 0.012 in Accra (Fig 2).

BLAST analyses showed that all coronaviruses detected in this study had pairwise sequence identity between 97.70% and 99.90% with the previously reported Eidolon bat coronavirus/Kenya/KY24/2006 of the genus *Betacoronavirus* (pairwise sequence identity ranged between 97.70% to 99.70% in Accra and between 98.10% to 99.90% in Morogoro).

### Precipitation

In Morogoro, the precipitation was low from September through February and in July, with the rainy season starting in March and ending in June. In Accra, the rainy season started in March and ended in August, and the dry season extended from October through February. The peak of the wet season paralleled the “weaning” period in both roosts (Fig 2).

### Statistical analysis

MCMC sampling diagnostics are provided in S4 File. Summaries of the Posterior Probability Distributions (PProDs) of models’ parameters are provided in S5 File. The LOOIC of the intercept-only model and the sine-cosine model were 795.462 and 764.9, respectively, suggesting a better fit of the latter, which incorporated seasonality. Some of the modeled monthly proportions of *E*. *helvum* shedding CoV (PpreDs) did not overlap: i) from October to January versus June and ii) from November to December versus May and July. The lack of overlap further supports CoV shedding seasonality based on the criteria established in the “Methods” section (Table 1).

**Table 1:** The lower and upper endpoints of the 95% Highest Posterior Density Interval (HPDI) of the predicted monthly proportion of *Eidolon helvum* shedding coronaviruses in Morogoro, Tanzania produced by a sine-cosine model with a period of 12 months and a single annual maximum and minimum.

Month	95% Highest Posterior Density Interval endpoints	
	Minimum	Maximum
August	0.052	0.196
September	0.031	0.144
October^1^	0.010	0.103
November^1^^,^^3^	0.010	0.093
December^1^^,^^3^	0.010	0.093
January^1^	0.010	0.103
February	0.031	0.144
March	0.052	0.196
April	0.072	0.227
May^4^	0.095	0.263
June^2^	0.113	0.278
July^4^	0.093	0.258

^1^ and ^2^ months have 95% HPDIs that do not overlap.

^3^ and ^4^ months have 95% HPDIs that do not overlap.

**Fig 3:**
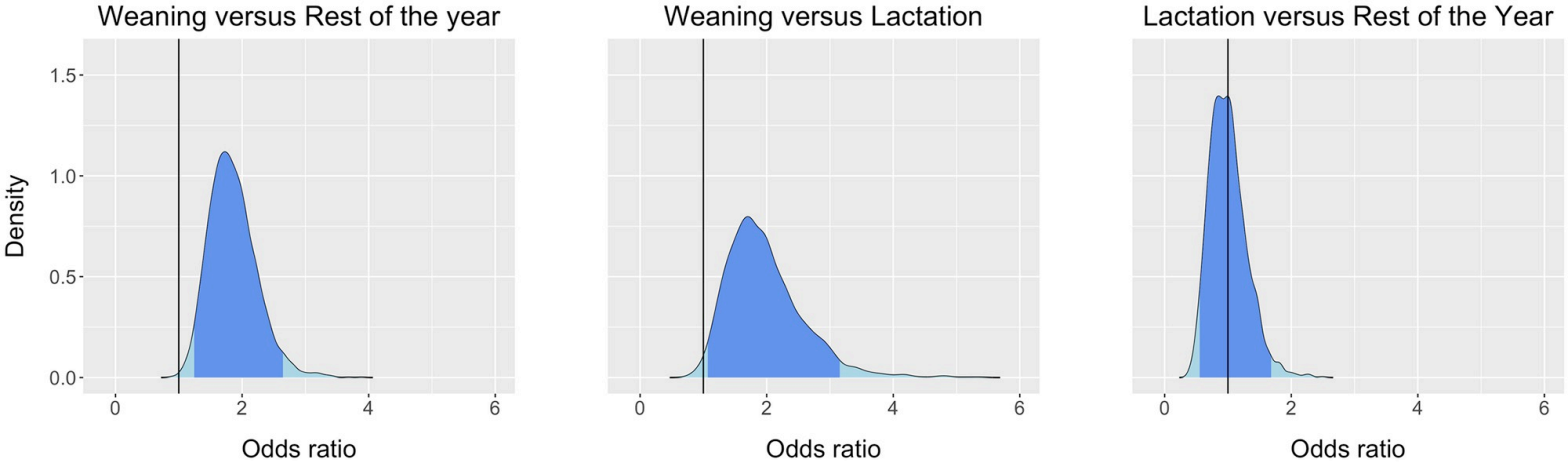
The Posterior Probability Distributions (light blue) and the corresponding 95% Highest Posterior Density Interval (blue) of the odds ratio for coronavirus shedding by *Eidolon helvum* in the roost at Morogoro Tanzania during the “weaning” period versus the “rest of the year”, during the “weaning” period versus the “lactation” period, and during the “lactation” period versus the “rest of the year”. The vertical black line indicates a neutral odds ratio with a value of one.

According to the “fixed model” 95% HPDI results, the odds of CoV shedding during the “weaning” period were between 1.24 and 2.65 times higher than in the “rest of the year”, and 1.06 to 3.16 times higher during the “weaning” period compared to the “lactation” period. Lastly, the model does not support differences between the “lactation” and “rest of the year” period (odds ratio covered the neutral value of 1; Fig 3).

The “hierarchical model” results showed higher odds of CoV shedding during the later months of the “weaning” period compared to the “rest of the year”, as well as heightened shedding during the peak of the “weaning” period versus the “lactation” period. Moreover, the reproductive period standard deviation PProD (σ_R_) tended to contain larger values compared to the month standard deviation PProD (σ_M_; S5 and S6 Files).

## Discussion

The straw-colored fruit-bat, *E*. *helvum*, is a key seed-disperser of Africa that has responded to land-use change by occupying trees in urban centers. This adaptation has led to intense human-bat interfaces across the continent. Coronaviruses in these bats are prevalent at these interfaces, presenting a challenge to reduce pathogen exposure in people while also supporting bat conservation.

To assess public health and conservation win-win solutions, we conducted a longitudinal study to assess coronavirus shedding dynamics in *E*. *helvum* urban colonies. This unique effort involved the testing of thousands of fecal samples that were collected on a monthly basis during an entire year, the inclusion of two urban roosts separated by more than four thousand km, the quantification of roost sizes across the study, and the inference of the reproductive seasons at both colonies.

The coronaviruses detected in both colonies had high pairwise sequence identity with the betacoronavirus Eidolon bat coronavirus/Kenya/KY24/2006 which has been found in *E*. *helvum* elsewhere in Africa [24, 26, 27, 34, 54]. This strain represents approximately 94% of all coronavirus detections in this species to date [55]. Eidolon bat coronavirus/Kenya/KY24/2006 has also been reported in the Chiropterans *Epomops franqueti*, *Megaloglossus woermanni*, *Mops condylurus*, *Rousettus aegyptiacus*, *Scotophilus dinganii*, *Tadarida sp*., and *Triaenops persicus* [55]. Until proven otherwise, there is no evidence to date that the specific coronaviruses detected in the studied bat colonies in Morogoro or Accra present a threat to people’s health and the RNA detected in this study through PCR does not necessarily equate to an infectious coronavirus. Nevertheless, its broad distribution inclusive of urban areas, warrants further study.

The overall proportion of positive feces was markedly lower in Accra despite equivalent sampling, storage, transport, and testing protocols. Low coronavirus detection has been previously reported in Ghanaian *E*. *helvum* roosts [56, 57]. We could not assess if roost-specific demography could explain these results because we did not capture animals and demographic data is limited and arguably biased [7].

In both roosts, our results support that coronavirus shedding is seasonal with a peak during the corresponding colony pup weaning season, regardless of the dramatically different roost sizes. In Morogoro, the peak roost size precedes the coronavirus detection peak, but the detection peak occurs before the peak population size in Accra. We observed that as the colony size increased also did the number of occupied trees, whilst roosting group size seemed to remain constant. The parallel change in bat populations and occupied trees with perceived constant group sizes may have yielded relatively similar contact rates over time and is consistent with the absence of an evident trend between roost size and CoV detection.

Coronavirus shedding seasonality with a peak in the weaning season has been reported in non-African bat species ([58–63] but see [64]). However, only point estimates of coronavirus positivity have been presented to date, except for three studies that provided confidence intervals but assuming statistical independence of the tested specimens regarding coronavirus presence [59, 62, 63]. The confidence intervals during each sampling event reported in [59, 62] overlapped, suggesting either a lack of statistical power or an actual difference in positivity over time. Further, these non-African studies reported inconsistent and small sample sizes, had variable time lags between sampling events, included only a single colony (leaving uncertainty about pattern consistency across roosts), or assumed independence of the specimens collected during the same sampling event. Our design allowed us to overcome these constraints and to statistically model coronavirus shedding to support the feasibility of seasonal management of human exposure risk to coronavirus shedding in densely-populated human areas.

The assumed coronavirus shedding seasonality has hypothetically been attributed to the waning of passively-acquired maternal antibodies in neonates [58]. Supporting this idea, coronavirus detection was more common during weaning periods and in non-adult bats [25, 34, 59, 61, 62, 65–67]. We did not directly observe pups as part of this non-invasive study, but our group and previous authors have observed them attached to lactating females in both studied colonies [19, 37], favoring the possibility of naïve juvenile influx as a driver of coronavirus infection during the weaning period. This influx could impact coronavirus transmission across the entire colony, leading to higher detection in adult bats during the weaning period as well [25, 65]. Higher detection during this season could also be multifactorial. For example, *E*. *helvum* mates during the weaning period [3, 18], which could also impact contact rates, susceptibility, and infectiousness of individuals, and consequently, virus transmission dynamics.

*Eidolon helvum* can migrate thousands of km and this species is proposed to be panmictic at <6,500 km across its continental African range [22]; however, migration routes and interconnectivity among distant colonies remain unknown. We did not aim to study the epidemiological relationships among the studied roosts and the impact of coronavirus dynamics in a roost upon the trends of other roosts remains unknown.

The logistical challenges to mitigate human viral exposure at urban bat colonies highlight the potential for evidence-based forecasting of high shedding seasons that could guide resource allocation. Although more research is needed to characterize the zoonotic potential of the coronaviruses hosted by *E*. *helvum* and to understand whether greater shedding is associated with higher probabilities of spillover, our results support that the resources available to prevent human coronavirus exposure at urban colonies could be more efficiently targeted for use during the high-shedding “weaning” period. During this period, access to roosts and surrounding areas could be limited, especially at the times of the day when bats are actively leaving and returning to their roots. Hunting and selling of bats could be seasonally banned to protect human health. Consumption could also be generally discouraged. In addition, human-use areas below roost trees could be adapted to protect people from bat droppings.

*E*. *helvum* is a key seed disperser for the currently highly-fragmented habitats of tropical Africa [16, 17], which makes their protection a conservation priority for the bats themselves, as well as the tropical plant species that they support. Therefore, management strategies that avoid culling are essential. Besides the ethical and welfare concerns, retaliatory killing has failed to reduce viral infection levels in bats, and this practice could lead to younger populations, favoring infection and shedding in roosts [68]. Mitigation of coronavirus exposure by seasonally altering human behavior also prevents roost perturbation during the ecologically-sensitive period when pups are born, nursed, and weaned.

We expect that our model-based definition of a high-shedding season will be applicable for roosts located in other urban centers of Africa [5, 7, 18, 19]. Previous data can support the prediction of the birth pulses and weaning periods over time and space for targeting mitigation interventions in other colonies [69].

## Conclusions

Straw-colored fruit bats (*E*. *helvum*) at the urban roosts of the 37 Military Hospital (Accra, Ghana, West Africa) and Kikundi Market–Nunge Court (Morogoro, Tanzania, East Africa) shed coronaviruses through feces not uniformly across the year but seasonally in association with the annual reproductive cycle of this species. In these two urban roosts, coronaviruses were found in a higher proportion of fecal samples during the corresponding annual weaning period.

These two urban roosts represent a main wildlife-human interface for conservation conflict but also for zoonotic pathogen transmission. Therefore, understanding the critical moments of coronavirus shedding to prevent spillover is key to elaborate win-win One Health solutions that promote the delivery of the ecosystems services provided by *E*, *helvum*, a prominent African seed-disperser, while safeguarding public health.

The consistency of the observed coronavirus shedding dynamics support that human exposure to urban *E*. *helvum* roosts should be limited when individuals smaller than the adult size are sighted (to establish the birth pulse-weaning period). This criterion can be applied in locations where sample collection and testing are hard to accomplish or where they are simply infeasible. Model-based establishment of reproductive seasons is a potential promising tool to apply this mitigation strategy across *E*. *helvum* range.

## Supporting information

S1 Fig Collection of fecal samples from plastic sheets set below specific trees occupied by *Eidolon helvum* in Morogoro. (PDF)

S1 File Other references reporting the detection of viral RNA and DNA and isolation of viruses from diverse viral taxonomic families including those with zoonotic species in *Eidolon helvum*. (PDF)

S2 File Models equations. (PDF)

S3 File MCMC sampling and sampling diagnostics details. (PDF)

S4 File Sampling diagnostic results. (PDF)

S5 File Summary of the posterior probability distributions of models parameters. (PDF)

S6 File Posterior probability distributions of the odds ratios of coronavirus shedding in *Eidolon helvum* in different months within different reproductive periods, and of the standard deviations of the reproductive period and month distributions. (PDF)
