# Supplementary material for: Seasonal shedding of coronavirus by straw-colored fruit bats at urban roosts in Africa
Source: PLoS One. 2022 Sep 15;17(9):e0274490. doi: 10.1371/journal.pone.0274490 (PMC9477308; doi:10.1371/journal.pone.0274490)

**S1 Fig. Collection of fecal samples from plastic sheets set below specific trees occupied by *Eidolon helvum* in Morogoro.** Picture by Dr. Abel Ekiri.

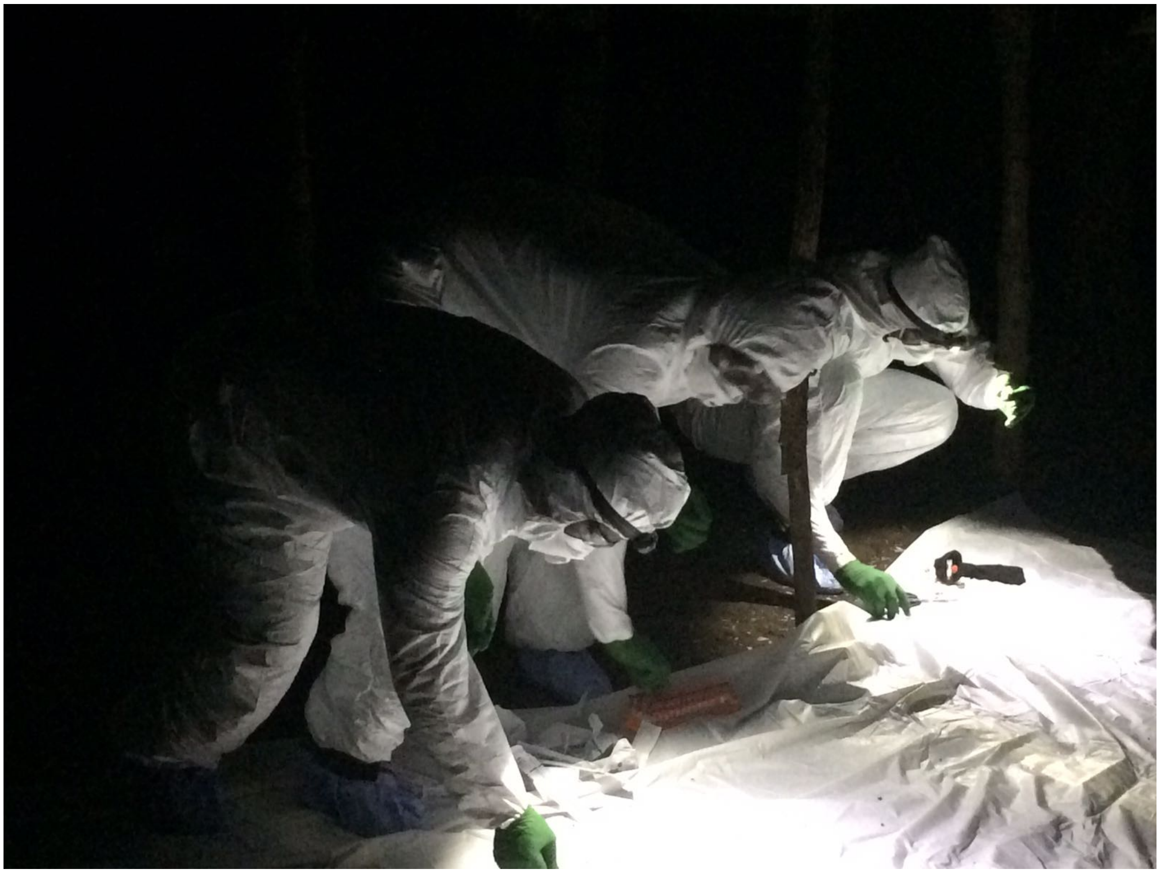

Supplement: S1 Fig — (PDF) [file pone.0274490.s001.pdf]
