# Supplementary material for: Seasonal shedding of coronavirus by straw-colored fruit bats at urban roosts in Africa
Source: PLoS One. 2022 Sep 15;17(9):e0274490. doi: 10.1371/journal.pone.0274490 (PMC9477308; doi:10.1371/journal.pone.0274490)
Supplement: S1 File — (PDF) [file pone.0274490.s002.pdf]

S1 File. Other references reporting the detection of viral RNA and DNA and isolation of viruses from diverse viral taxonomic families including those with zoonotic species in *Eidolon helvum*.

1. L. R. Boulger, J. S. Porterfield, Isolation of a virus from Nigerian fruit bats. *Trans. R. Soc. Trop. Med. Hyg.* **52**, 421–424 (1958).

2. G. E. Kemp, G. Le Gonidec, N. Karabatsos, A. Rickenbach, C. B. Cropp, IFE: a new African orbivirus isolated from *Eidolon helvum* bats captured in Nigeria, Cameroon and the Central African Republic. *Bull. Soc. Pathol. Exot. Filiales* **81**, 40–48 (1988).

3. M. D. Esona, *et al.*, Reassortant group A rotavirus from straw-colored fruit bat (*Eidolon helvum*). *Emerg. Infect. Dis.* **16**, 1844–1852 (2010).

4. K. S. Baker, *et al.*, Novel, potentially zoonotic paramyxoviruses from the African straw-colored fruit bat *Eidolon helvum*. *J. Virol.* **87**, 1348–1358 (2013).

5. K. S. Baker, *et al.*, Metagenomic study of the viruses of African straw-coloured fruit bats: detection of a chiropteran poxvirus and isolation of a novel adenovirus. *Virology* **441**, 95–106 (2013).

6. C. Conrardy, *et al.*, Molecular detection of adenoviruses, rhabdoviruses, and paramyxoviruses in bats from Kenya. *Am. J. Trop. Med. Hyg.* **91**, 258–266 (2014).

7. T. Binger, *et al.*, A novel rhabdovirus isolated from the straw-colored fruit bat *Eidolon helvum*, with signs of antibodies in swine and humans. *J. Virol.* **89**, 4588–4597 (2015).

8. C. M. Freuling, *et al.*, Lagos bat virus transmission in an *Eidolon helvum* bat colony, Ghana. *Virus Res.* **210**, 42–45 (2015).

9. S. Leopardi, *et al.*, The close genetic relationship of lineage D Betacoronavirus from Nigerian and Kenyan straw-colored fruit bats (*Eidolon helvum*) is consistent with the existence of a single epidemiological unit across sub-Saharan Africa. *Virus Genes* **52**, 573–577 (2016).

10. C. Waruhiu, *et al.*, Molecular detection of viruses in Kenyan bats and discovery of novel astroviruses, caliciviruses and rotaviruses. *Virol. Sin.* **32**, 101–114 (2017).

11. C. K. Yinda, *et al.*, Novel highly divergent sapoviruses detected by metagenomics analysis in straw-colored fruit bats in Cameroon. *Emerg. Microbes Infect.* **6**, e38 (2017).

12. G. E. Kemp, O. R. Causey, D. L. Moore, A. Odelola, A. Fabiyi, Mokola Virus. *Am. J. Trop. Med. Hyg.* **21**, 356–359 (1972).

13. D. T. S. Hayman, *et al.*, Long-term survival of an urban fruit bat seropositive for Ebola and Lagos bat viruses. *PLoS One* **5**, e11978 (2010).

14. E. Wright, *et al.*, Virus neutralising activity of African fruit bat (Eidolon helvum) sera against emerging lyssaviruses. *Virology* **408**, 183–189 (2010).

15. M. A. Müller, *et al.*, Evidence for widespread infection of African bats with Crimean-Congo hemorrhagic fever-like viruses. *Sci. Rep.* **6**, 26637 (2016).

16. L. N. Kalemba, *et al.*, Exposure to Lyssaviruses in bats of the Democratic Republic of the Congo. *J. Wildl. Dis.* **53**, 408–410 (2017).
