# Supplementary material for: Seasonal shedding of coronavirus by straw-colored fruit bats at urban roosts in Africa
Source: PLoS One. 2022 Sep 15;17(9):e0274490. doi: 10.1371/journal.pone.0274490 (PMC9477308; doi:10.1371/journal.pone.0274490)
Supplement: S2 File — (PDF) [file pone.0274490.s003.pdf]

S2 File. Models Equations.

- Intercept model

$$\boldsymbol{log\left(\frac{p_i}{1 - p_i}\right) = \alpha^o}$$

- Sine - cosine model equation:

$$\boldsymbol{log\left(\frac{p_i}{1 - p_i}\right) = \alpha^0 + \beta^{sine} * sine(2 * \pi * \frac{day_i}{365}) + \beta^{cos} * cos(2 * \pi * \frac{day_i}{365})}$$

- Fixed effects model equation:

$$\boldsymbol{log\left(\frac{p_i}{1 - p_i}\right) = \beta^0 + \beta^{weaning} * weaning_i + \beta^{lactation} * lactation_i}$$

- Hierarchical model equation:

$$log\left(\frac{p_i}{1 - p_i}\right) = \alpha_{m[i]}^{month\_repro}$$

where

$$\alpha_{m[i]}^{month\_repro} = \alpha_{r[m]}^{reproductive\_period} + \alpha_m^{month}$$ *for m = 1, 2, …, 12; and r = 1, 2, 3*

and

$$a_m^{month} \sim Normal(0, \sigma_M)$$

$$a_r^{reproductive\_period} \sim Normal(0, \sigma_R)$$

Finally,

$$\sigma_M \sim Half - Cauchy(0, 2)$$

$$\sigma_R \sim Half - Cauchy(0, 2)$$

Here p_i is the probability of coronavirus detection in the ith *Eidolon helvum* fecal sample; day_i is the index of the day of the year (1 to 365) when the ith *Eidolon helvum* fecal sample was collected; $weaning_i$ and $lactation_i$ indicate whether the ith *Eidolon helvum* fecal sample was collected during the "weaning" or "lactation" period, respectively; α^0 is the intercept, β^0, β^{sine}, β^{cos}, $\beta^{weaning}$, and $\beta^{lactation}$ are the coefficients for the intercept, the sine and cosine terms, and the "weaning" and "lactation" periods, respectively. Finally, $\alpha_{m[i]}^{month\_repro}$ is the coefficient for the ith *Eidolon helvum* fecal sample collected during month *m* within the reproductive period *r*, α_m^{month} is the coefficient for the month *m* within the reproductive period *r*, and $\alpha_{r[m]}^{reproductive\_period}$ is the coefficient for the reproductive period *r*. The terms σ_M and σ_R are the standard deviation of reproductive period and month distributions.
