# Supplementary material for: Seasonal shedding of coronavirus by straw-colored fruit bats at urban roosts in Africa
Source: PLoS One. 2022 Sep 15;17(9):e0274490. doi: 10.1371/journal.pone.0274490 (PMC9477308; doi:10.1371/journal.pone.0274490)
Supplement: S3 File — (PDF) [file pone.0274490.s004.pdf]

## S3 File. MCMC sampling and sampling diagnostics details.

*Normal* (0, 1) priors were assigned for all model coefficients. Priors for random intercepts for months and reproductive periods were assigned as *Normal*(0, $\sigma_M$) and *Normal*(0, $\sigma_R$) with hyperpriors for $\sigma_M$ and $\sigma_R$ assigned as *Half-Cauchy*(0, 2). The PProdDs were obtained by sampling in parallel from 4 MCMC chains for 5,000 iterations each following a 5 thousand iteration warm-up and thinning of 5 for a total of 4,000 saved samples. Convergence was assessed by the Gelman-Rubin statistic (1) and graphically evaluated using trace plots.

1. A. Gelman, D. B. Rubin, Inference from Iterative Simulation Using Multiple Sequences. Stat. Sci. 7, 457–472 (1992).
