# Supplementary material for: Seasonal shedding of coronavirus by straw-colored fruit bats at urban roosts in Africa
Source: PLoS One. 2022 Sep 15;17(9):e0274490. doi: 10.1371/journal.pone.0274490 (PMC9477308; doi:10.1371/journal.pone.0274490)
Supplement: S4 File — (PDF) [file pone.0274490.s005.pdf]

## S4 File. Sampling diagnostics results.

Across models, the Markov Chain Monte Carlo chains had Gelman-Rubin statistics ≤ 1.003 and the trace plots indicated convergence as well (see below). The effective number of samples for the intercept terms, fixed terms, random terms, and the corresponding between-groups (month and reproductive period) standard deviations were larger than twice the number of chains multiplied by five, as previously recommended (1).

**Fig S4-1. Trace plot for the intercept term. Intercept-only model.**

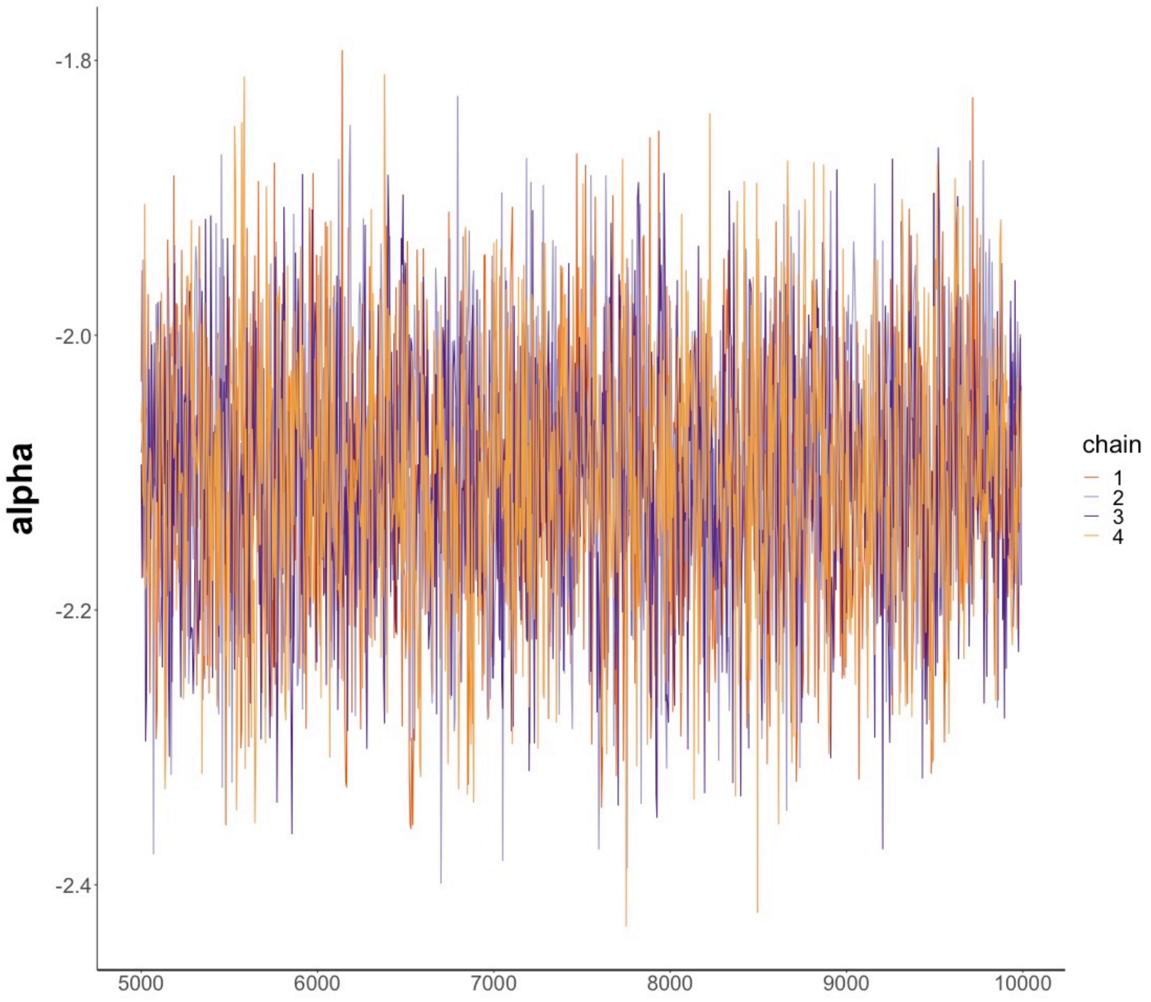

**Fig S4-2. Trace plot for the intercept term. Sine-cosine model.**

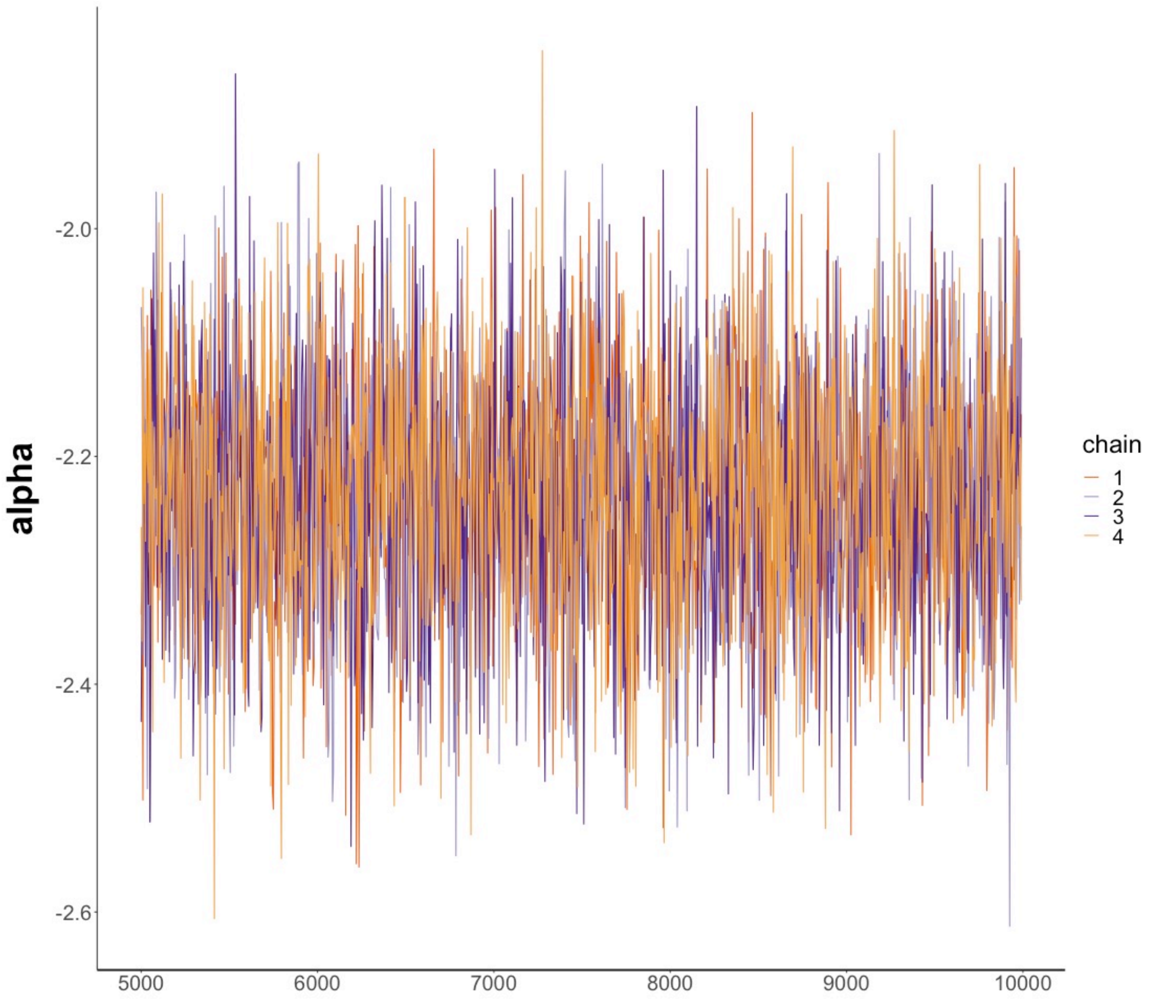

**Fig S4-3. Trace plot for the $\beta^{sine}$ term. Sine-cosine model.**

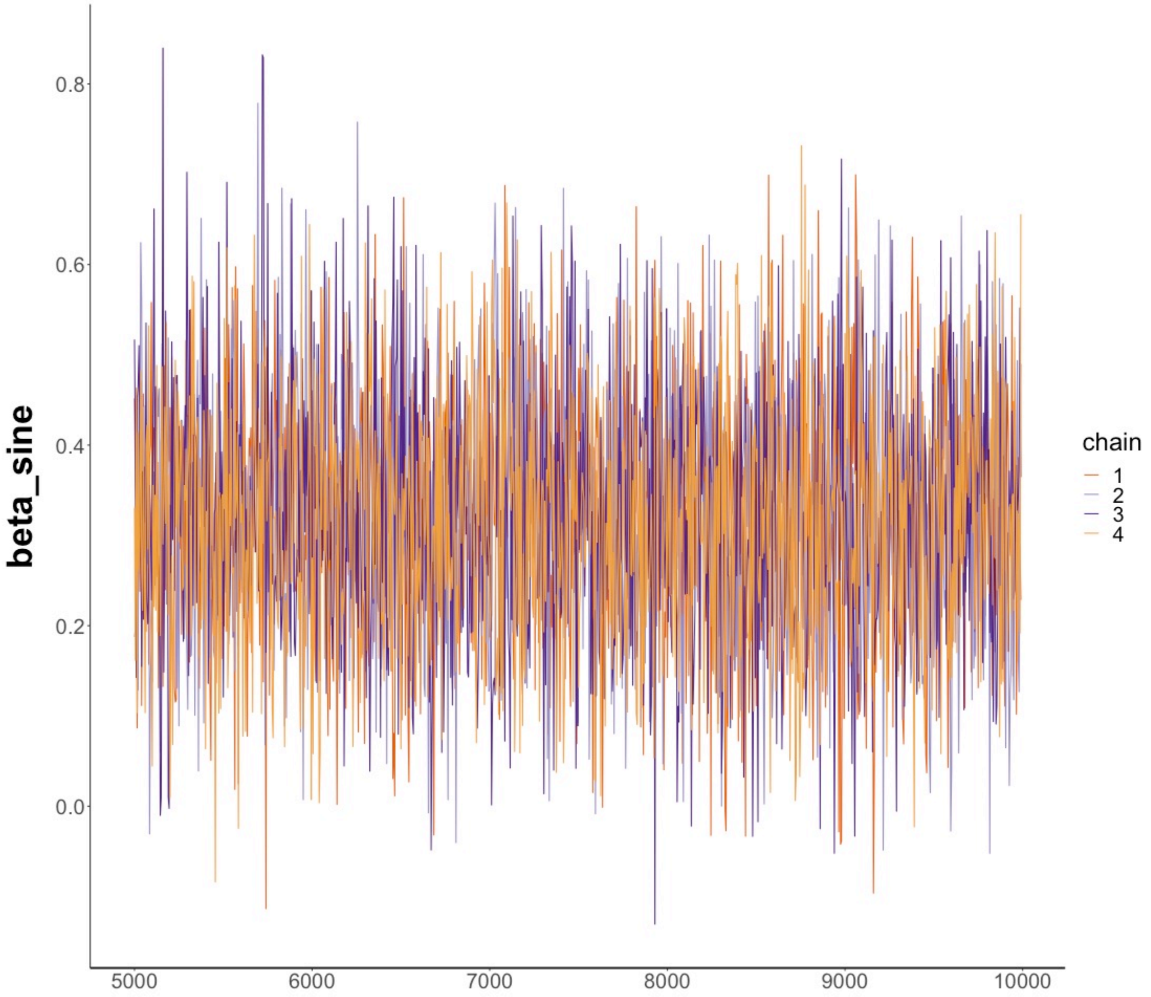

**Fig S4-4. Trace plot for the $\beta^{cos}$ term. Sine-cosine model.**

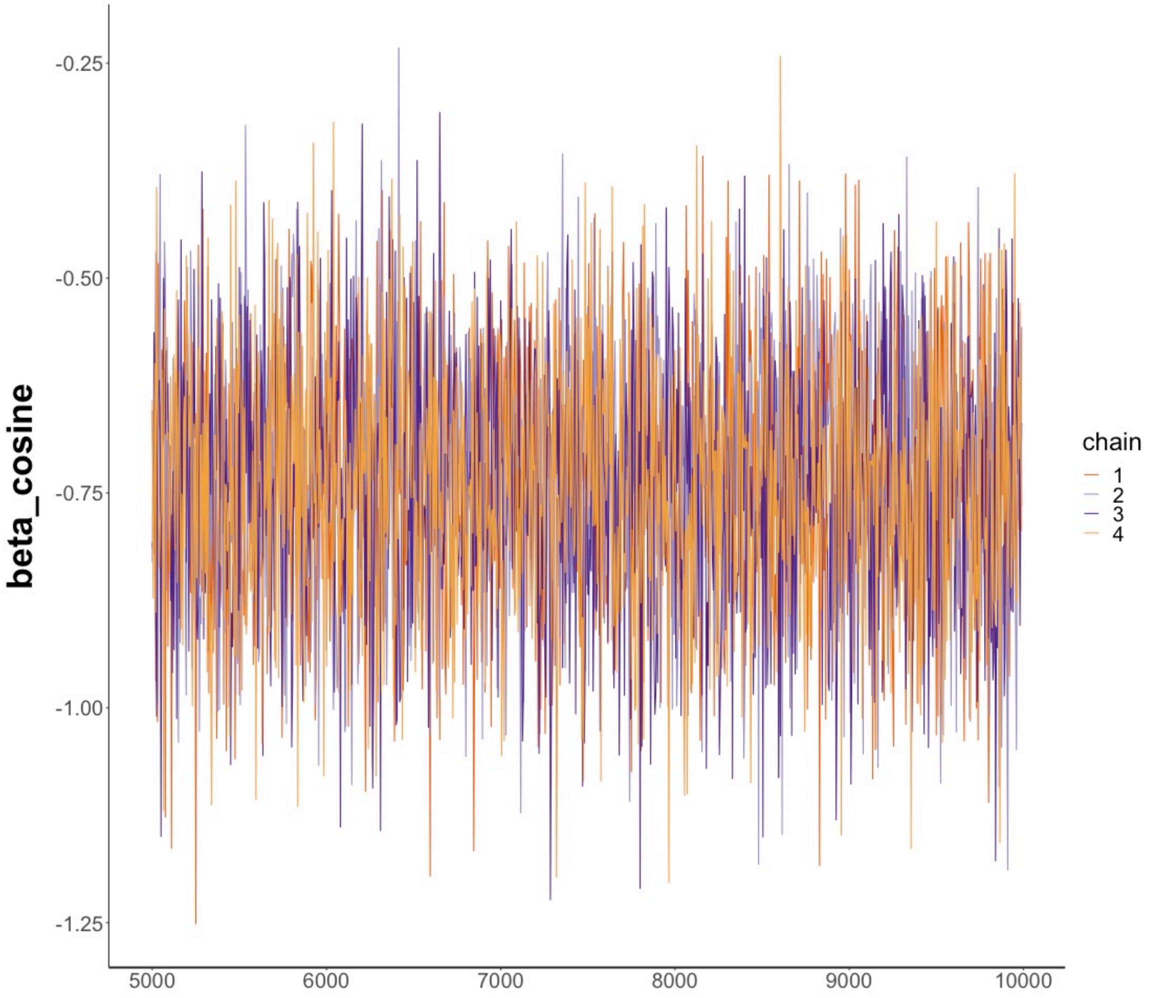

**Fig S4-5. Trace plot for the intercept  term. Fixed-effects model.**

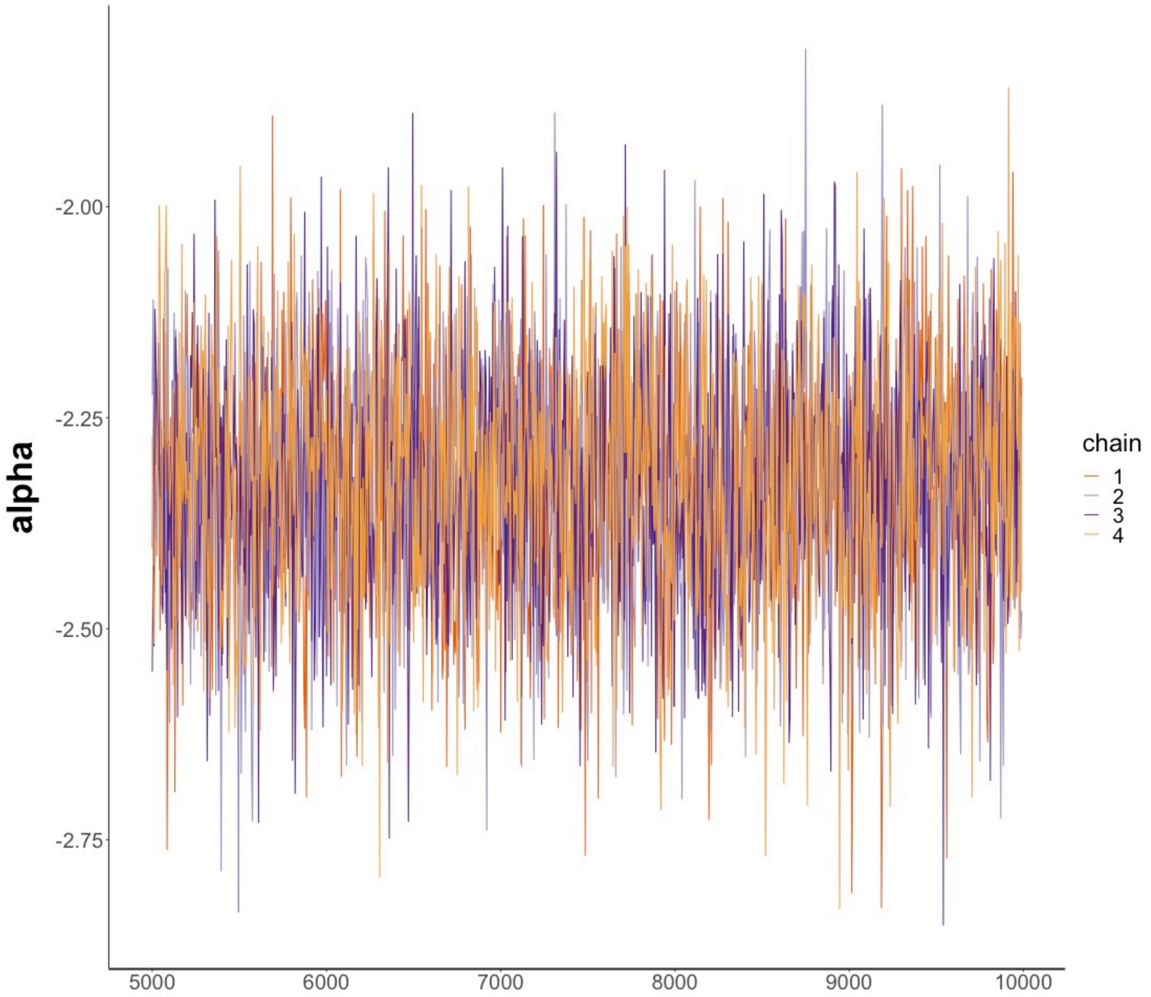

**Fig S4-6. Trace plot for the $\beta^{weaning}$ term. Fixed-effects model.**

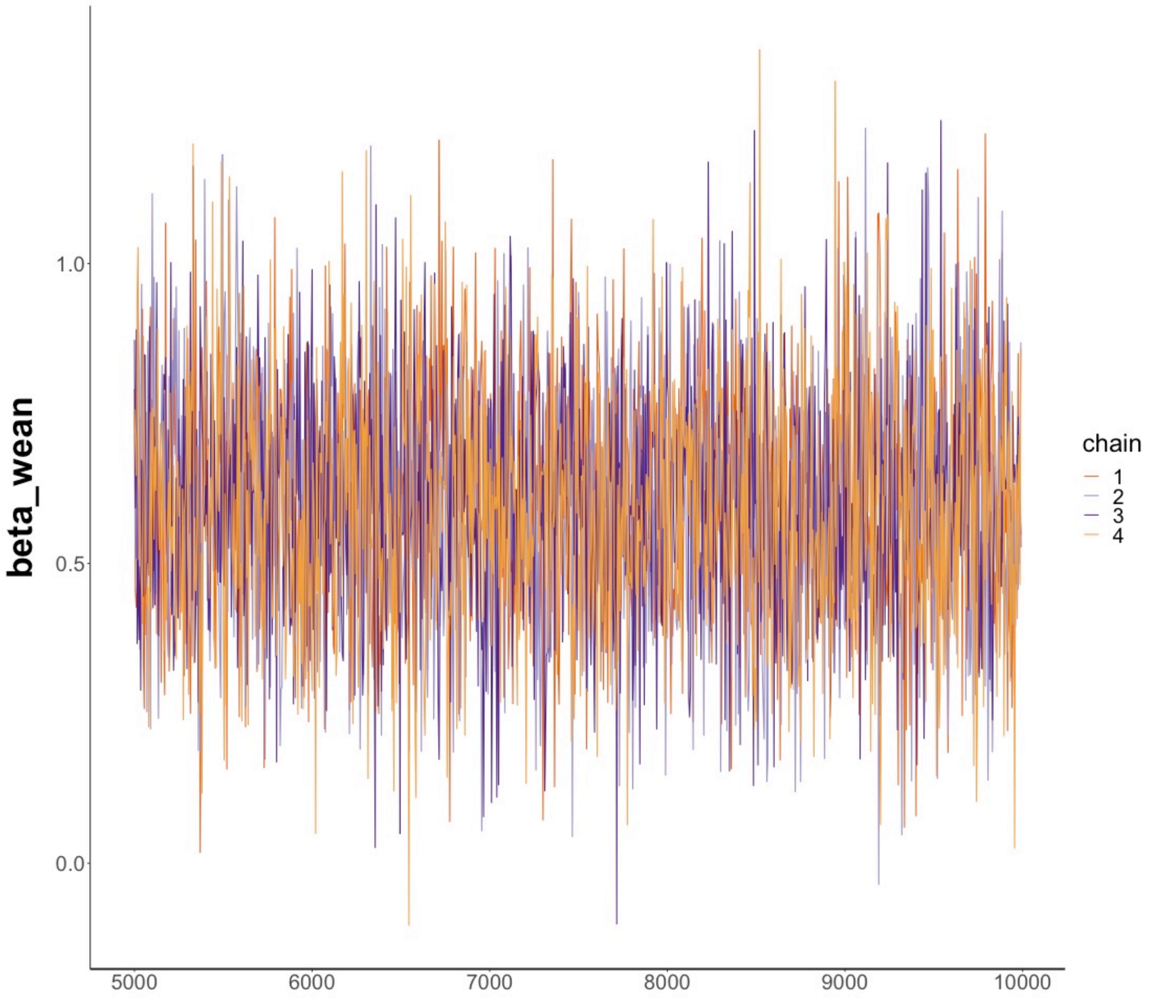

**Fig S4-7. Trace plot for the $\beta^{lactation}$ term. Fixed-effects model.**

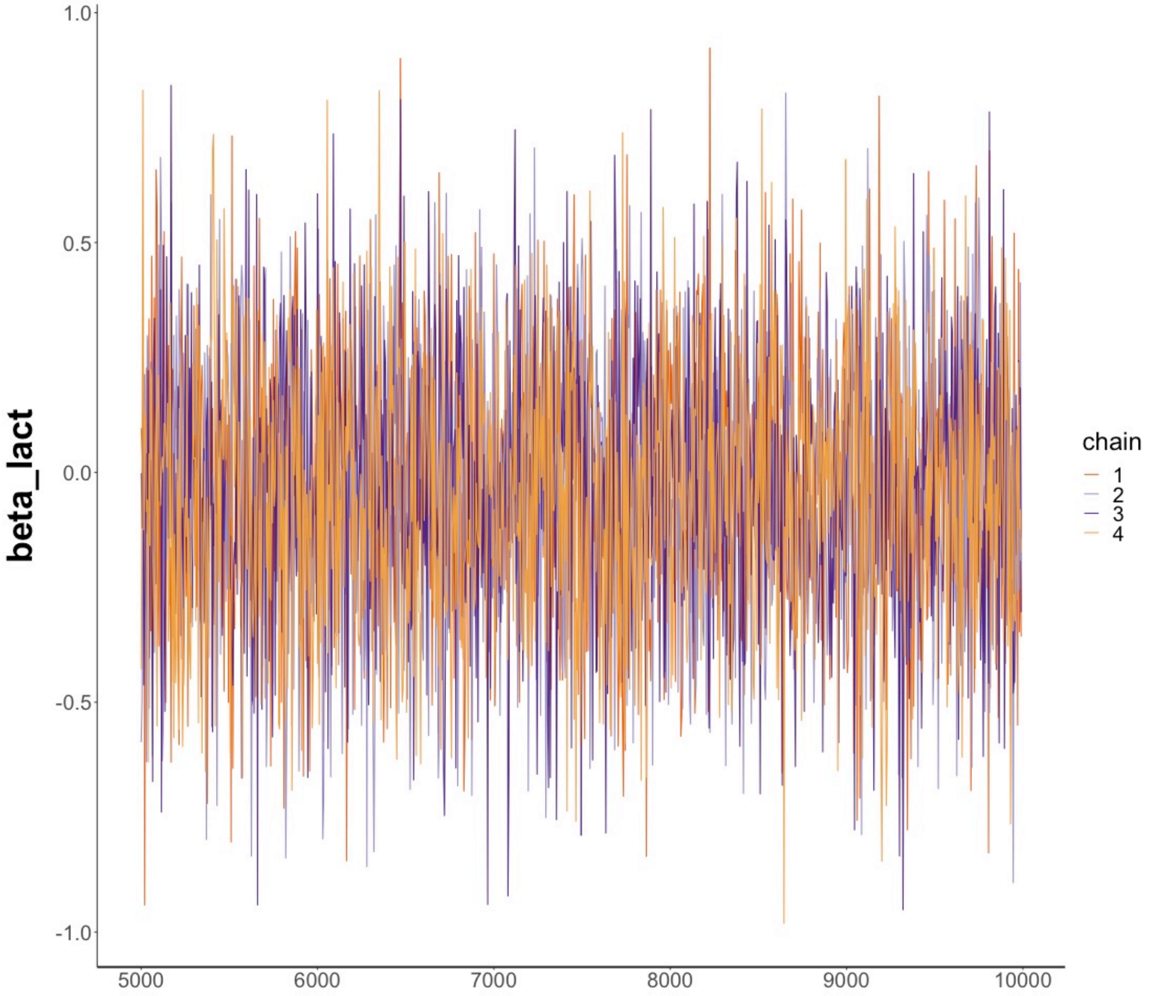

**Fig S4-8 Fig. Trace plot for the $\alpha_r^{reproductive\_period}$ terms. Hierarchical model.** The left, middle, and right panels show the trace plots for the "rest of the year", "lactation", and "weaning" periods, respectively.

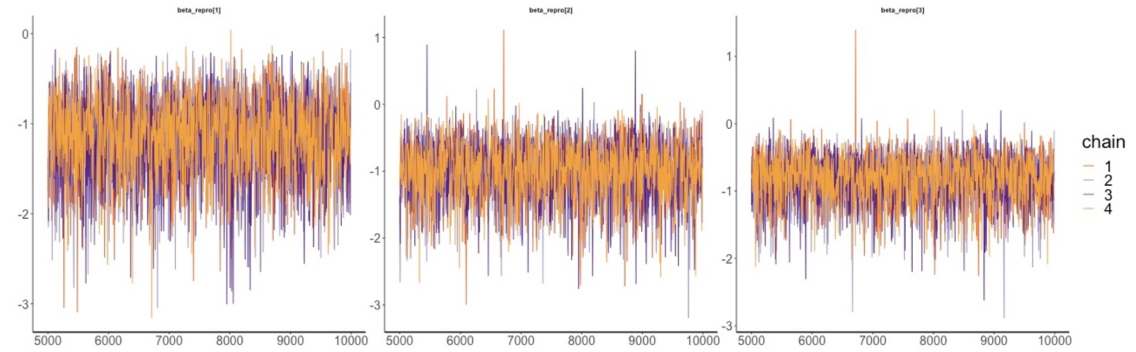

**Fig S4-9 Fig. Trace plot for the $\alpha_m^{month}$ terms. Hierarchical model**. The panels show the trace plots for the month parameters starting in August (top left), December (middle left), and April (bottom left).

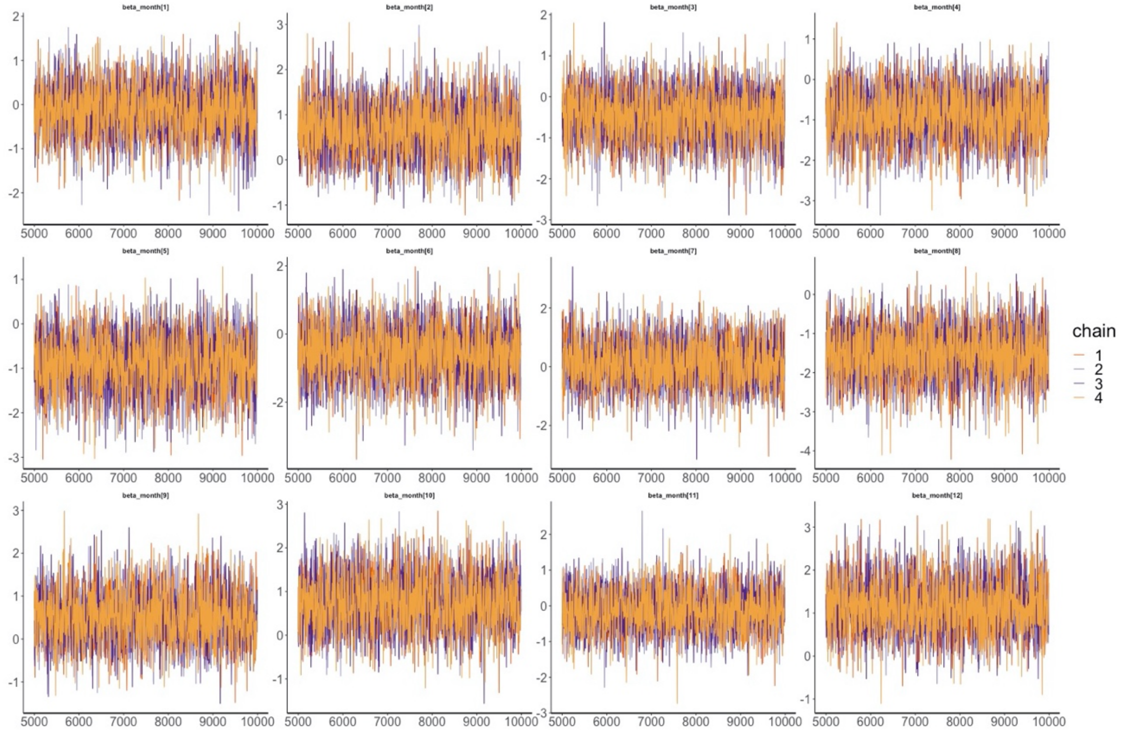

**Fig S4-10 Fig. Trace plot for the $\sigma_M$ term. Hierarchical model.**

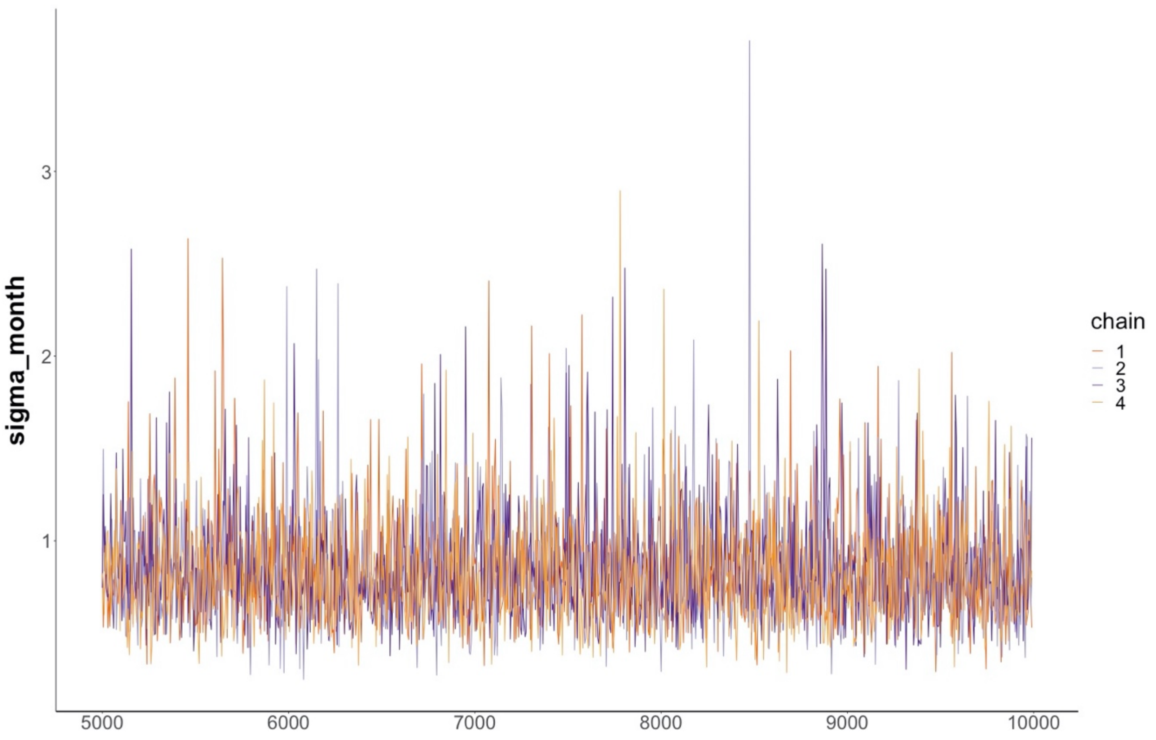

**Fig S4-11. Trace plot for the $\sigma_M$ term. Hierarchical model.**

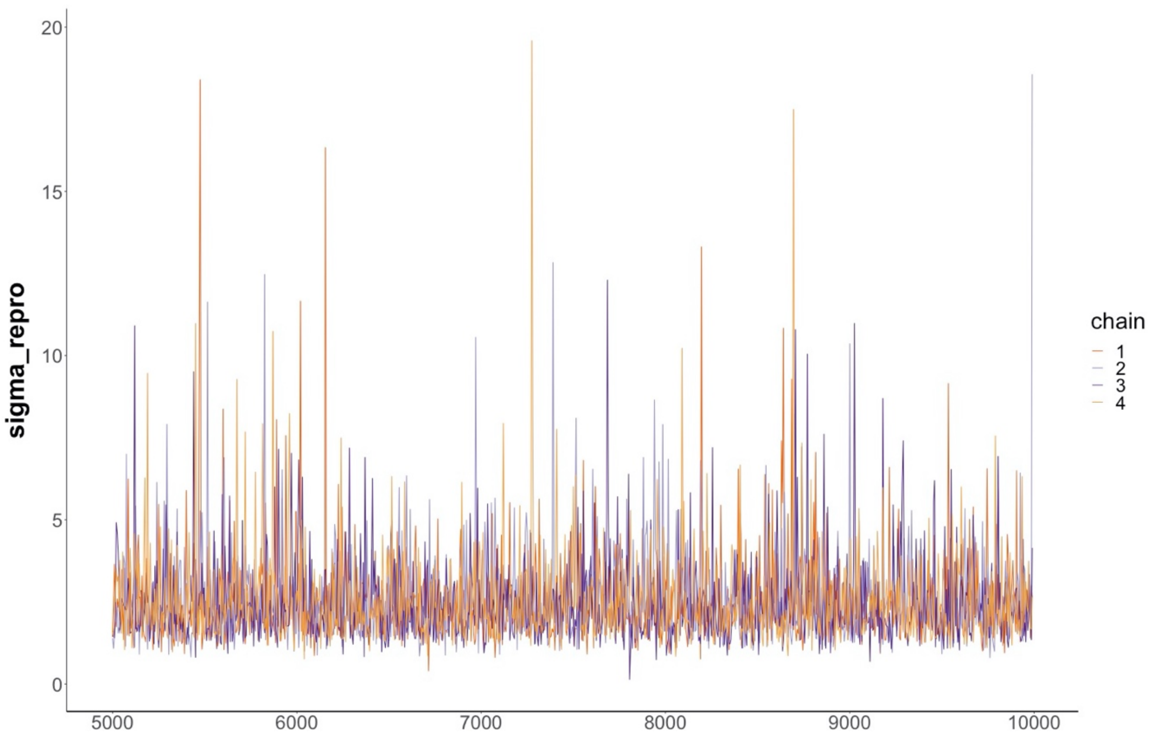



1. A. Gelman, D. B. Rubin, Inference from Iterative Simulation Using Multiple Sequences. Stat. Sci. 7, 457–472 (1992).
