# Supplementary material for: Seasonal shedding of coronavirus by straw-colored fruit bats at urban roosts in Africa
Source: PLoS One. 2022 Sep 15;17(9):e0274490. doi: 10.1371/journal.pone.0274490 (PMC9477308; doi:10.1371/journal.pone.0274490)
Supplement: S5 File — (PDF) [file pone.0274490.s006.pdf]

S5 File. Summary of the posterior probability distributions of models parameters.

Table S5-1. Summary of the Posterior Probability Distribution of the intercept-only model parameter.

Parameter	Mean	Standard deviation	95% Highest Posterior Density Interval endpoints	
			Minimum	Maximum
α^0	-2.104	0.095	-2.296	-1.930

Table S5-2. Summary of the Posterior Probability Distributions of the sine-cosine logistic model parameters.

Parameter	Mean	Standard deviation	95% Highest Posterior Density Interval endpoints	
			Minimum	Maximum
α^{0}	-2.235	0.107	-2.442	-2.018
β^{sine}	0.321	0.136	0.058	0.586
β^{cosine}	-0.740	0.146	-1.016	-0.453

Table S5-3. Summary of the Posterior Probability Distributions of the fixed-effects model parameters.

Parameter	Mean	Standard deviation	95% Highest Posterior Density Interval endpoints	
			Minimum	Maximum
α^{0}	-2.328	0.140	-2.601	-2.056
$\beta^{weaning}$	0.598	0.196	0.213	0.973
$\beta^{lactation}$	-0.038	0.287	-0.596	0.521

Table S5-4. Summary of the Posterior Probability Distributions of the hierarchical model parameters.

Parameter	Mean	Standard deviation	95% Highest Posterior Density Interval endpoints	
			Minimum	Maximum
$\alpha_{rest\ of\ the\ year}^{reproductive\_period}$	-1.164	0.461	-2.094	-0.363
$\alpha_{lactation}^{reproductive\_period}$	-1.005	0.448	-1.862	-0.185
$\alpha_{weaning}^{reproductive\_period}$	-0.828	0.365	-1.545	-0.181
α_{August}^{month}	-0.118	0.586	-1.289	1.028
$\alpha_{September}^{month}$	0.661	0.581	-0.428	1.823
$\alpha_{October}^{month}$	-0.461	0.611	-1.674	0.72
$\alpha_{November}^{month}$	-0.876	0.638	-2.124	0.348
$\alpha_{December}^{month}$	-0.875	0.634	-2.245	0.268
$\alpha_{December}^{month}$	-0.562	0.78	-2.137	0.892
$\alpha_{February}^{month}$	0.121	0.782	-1.317	1.698
α_{March}^{month}	-1.495	0.66	-2.859	-0.282
α_{April}^{month}	0.497	0.596	-0.648	1.703
α_{May}^{month}	0.74	0.606	-0.493	1.881
α_{June}^{month}	-0.123	0.586	-1.218	1.05
α_{July}^{month}	1.136	0.606	0.064	2.424
σ_R	2.544	1.402	0.838	4.866
σ_M	0.843	0.295	0.382	1.422
