# Supplementary material for: Seasonal shedding of coronavirus by straw-colored fruit bats at urban roosts in Africa
Source: PLoS One. 2022 Sep 15;17(9):e0274490. doi: 10.1371/journal.pone.0274490 (PMC9477308; doi:10.1371/journal.pone.0274490)

S6 File. Posterior probability distributions of the odds ratios of coronavirus shedding in *Eidolon helvum* in different months within different reproductive periods, and of the standard deviations of the reproductive period and month distributions.

Fig S6-1. Posterior probability distributions of the odds ratios of coronavirus shedding in *Eidolon helvum* when comparing month m within reproductive period r and month m' within reproductive period r'. The darkest blue shows the density within the 95% Highest Posterior Density Interval. The vertical lines show the odds ratio with value 1.

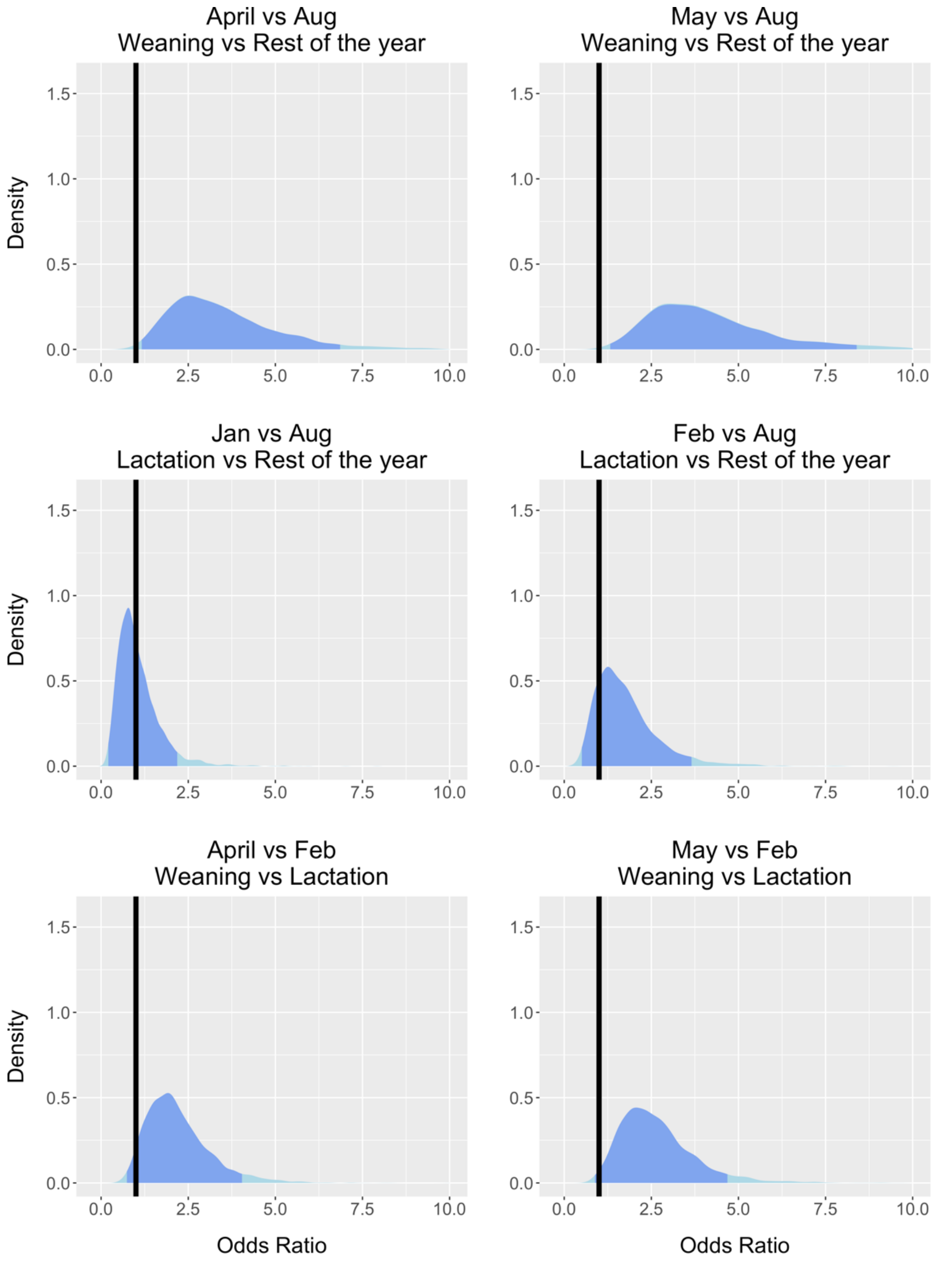

Fig S6-2. Posterior probability distributions of the reproductive period-level standard deviation (σ_R; pale red) and the month-level standard deviation (σ_M; pale green).

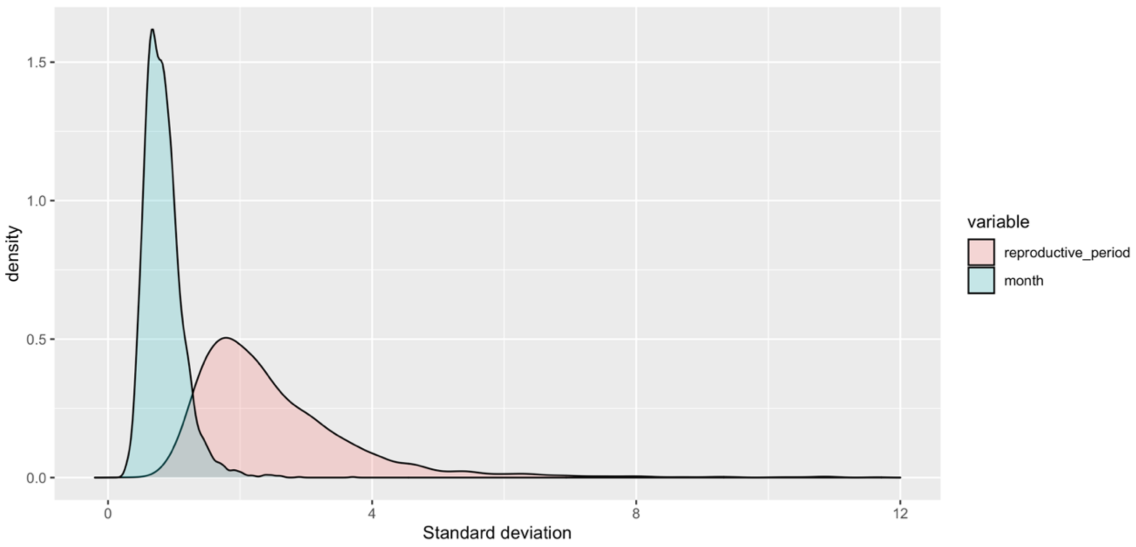

Supplement: S6 File — (PDF) [file pone.0274490.s007.pdf]
